# Recent developments in surface-enhanced Raman spectroscopy-based immuno (apta) assays for cardiac troponin detection: prospects for point-of-care applications

**DOI:** 10.1007/s12551-025-01381-z

**Published:** 2025-12-08

**Authors:** Sreejith K P, Murukeshan Vadakke Matham, Santhosh Chidangil

**Affiliations:** 1 Centre of Excellence for Biophotonics, Manipal Institute of Applied Physics, Manipal Academy of Higher Education, Manipal, Karnataka 576104 India; 2 Centre for Optical and Laser Engineering, School of Mechanical and Aerospace Engineering, Nanyang Technological University, 50 Nanyaang Avenue, Singapore, 639798 Singapore

**Keywords:** Surface-enhanced Raman spectroscopy (SERS), Cardiac troponin, Immunoassay, Point-of-care testing

## Abstract

Cardiac troponin (cTn) is widely recognized as the gold standard for the diagnosis of acute myocardial infarction (AMI), a leading cause of mortality worldwide. Point-of-care testing (POCT) for cTn necessitates rapid turnaround, cost-effectiveness, user-friendly operation, and portability, along with the highest level of accuracy to support timely and effective clinical decision-making. Surface-enhanced Raman spectroscopy (SERS) based assays possess several features suitable for POCT, including the flexibility to integrate with innovative platforms such as microfluidic systems and paper-based devices. In this review, we discuss the recent developments in SERS-based immuno (apta) assays for cTn detection, with a focus on detection strategies, employed platforms, and their feasibility for POCT. This review also outlines the improvements required in existing assays to meet POCT requirements, aiding in the design of future diagnostic assays.

## Introduction

The world’s leading cause of illness and mortality is cardiovascular disease (CVD). As to the World Health Organization (WHO), CVDs accounted for 17.9 million deaths worldwide in 2019 (World Health Organization (n.d.)), with heart attacks and strokes accounting for 85% of these deaths. Among the various types of CVDs, acute myocardial infarction (AMI), commonly known as a heart attack, is one of the serious and time-sensitive medical conditions (Pedrero et al. 2014; Virani et al. 2020; John et al. 2022b). There is a sudden interruption of blood flow in an acute myocardial infarction (AMI) due to the rupture of an atherosclerotic plaque, which subsequently leads to blood clots in the coronary artery. This, in turn, leads to ischemia (a lack of oxygen) and damages the myocardium (Aydin et al. 2019). This condition requires rapid and accurate diagnosis and treatment to reduce mortality and improve patient outcomes (Virani et al. 2020). The use of cardiac biomarkers in diagnosing CVDs, particularly AMI, has significantly advanced over the years. These biomarkers are released into the blood circulation following a myocardial injury and follow a noticeable rise and fall pattern. These tools provide early diagnosis and the prognosis of the condition (Yuan et al. 2021).

The diagnosis of AMI is based on the clinical presentation, electrocardiograms (ECGs), and variations in certain cardiac biomarker levels (Nagele 2020). The key markers, such as cardiac troponins (cTn), myoglobin, and CK-MB, are commonly used to diagnose AMI (Aydin et al. 2019). Among these biomarkers, cardiac troponins, especially cardiac troponin-I (cTnI) and cardiac troponin-T (cTnT), are recognized as the gold standards in detecting AMI. The continuous measurement of these troponins offers high specificity and sensitivity, and their release kinetics, especially in the early stages of myocardial damage (Aydin et al. 2019; Shi et al. 2020; Yuan et al. 2021). It is released into circulation within hours of myocardial injury and remains detectable for several days. This makes it a valuable tool for early diagnosis and detecting reinfarction, especially when serial measurements are taken (e.g., at 0/1-h or 0/2-h intervals). However, these values must be interpreted in the context of the patient’s clinical history, ECG, monitoring, and disease management (Laugaudin et al. 2016; Collet et al. 2021). Furthermore, its cardio specificity enables cTnI to differentiate between myocardial ischemia phases and other cardiac conditions such as myocarditis, heart failure, and stable angina, especially in relapsed patients (Tandon et al. 2019; Chaulin 2021b). By enabling earlier diagnosis and more accurate risk assessment in patients presenting with chest pain, the discovery of cTns has revolutionized the clinical management of AMI. However, for the detection of cTns, highly sensitive and precise analytical methods are required due to the extremely low concentrations released into the bloodstream during the early stages of myocardial damage (Michailovich Chaulin 2023). In the clinical laboratory setting, troponins are estimated mainly from blood samples using immunoassays, including enzyme-linked immunosorbent assays (ELISA), chemiluminescent immunoassays, fluorescence analysis, gold-labeled immunoassays, and immunoturbidimetry (Chen et al. 2023b). However, early-generation immunoassays lack sufficient sensitivity, often causing a delay in the diagnosis of myocardial injury, since measurable increases in troponin levels appeared relatively late following cardiomyocyte death. Furthermore, these laboratory-based assays require specialized equipment, skilled technicians, and frequently have turnaround times ranging from 20 to 90 min, which can impede clinical decision-making in acute settings (Hoofnagle and Wener 2009; Mair and Hammarsten 2023). Over time, troponin assays have evolved from low- and moderately sensitive methods to high-sensitivity (hs-cTn) and ultrasensitive immunoassays, significantly improving analytical performance and allowing for earlier detection of myocardial injury (Michailovich Chaulin 2023). On the other hand, Point-of-Care (POC) technologies represent a significant advancement for the rapid assessment of AMI outside centralized laboratory settings. Existing POC devices include TriageTrue hs-cTnI and PATHFAST hs-cTnI (a chemiluminescent enzyme immunoassay for the in vitro quantitative determination of cTnI in human anticoagulated whole blood and plasma on the PATHFAST analyzer). But the analytical and clinical performance of POC devices should be enhanced to match that of central laboratory assays (Thulin et al. 2023). However, they are rapidly advancing through the integration of wearable devices, smartphones, microfluidic systems, and paper-based technologies (Hu et al. 2016; Vasantham et al. 2020; Lin et al. 2021).

Surface-enhanced Raman spectroscopy (SERS) has consistently been at the forefront of clinical diagnostic technologies, owing to its strong potential for integration into point-of-care testing (POCT) devices. For the detection of cTn, commercially available POCT devices mainly utilize fluorescence, colorimetric, and chemiluminescence-based detection methods (Low et al. 2020). However, the limit of detection (LOD) achieved by many SERS-based assays for biomarker detection is significantly lower than that of colorimetric and fluorescent techniques (Fu et al. 2016; Wang et al. 2017a). SERS has also been demonstrated as an effective tool for multiplex detection of multiple analytes, owing to its much higher spectral resolution compared to fluorescence-based methods (Lee et al. 2014). Unlike fluorescence, SERS is nearly unaffected by photobleaching and quenching, producing stable signals that enable repeated measurements to minimize experimental errors (Ilyas et al. 2023). Further, SERS offers several important features for biomarker detection, including ultra-high sensitivity, rapid detection, relatively low cost, large dynamic range, simple pretreatment steps, and portability. In addition, portable Raman equipment is adaptable to a wide range of technologies, including paper devices, microfluidics, and optical fiber without complicating its operation (Marks et al. 2017; Lima et al. 2025).

In this review, we discuss the progress in SERS-based immunoassay and aptamer assay strategies for the detection and quantification of cTn (especially cTnI). The focus is on key parameters such as detection platforms, assay components, immunoreaction, LOD, immunoreaction time, and their suitability for meeting POCT requirements.

## Point-of-care testing

**Fig. 1:**
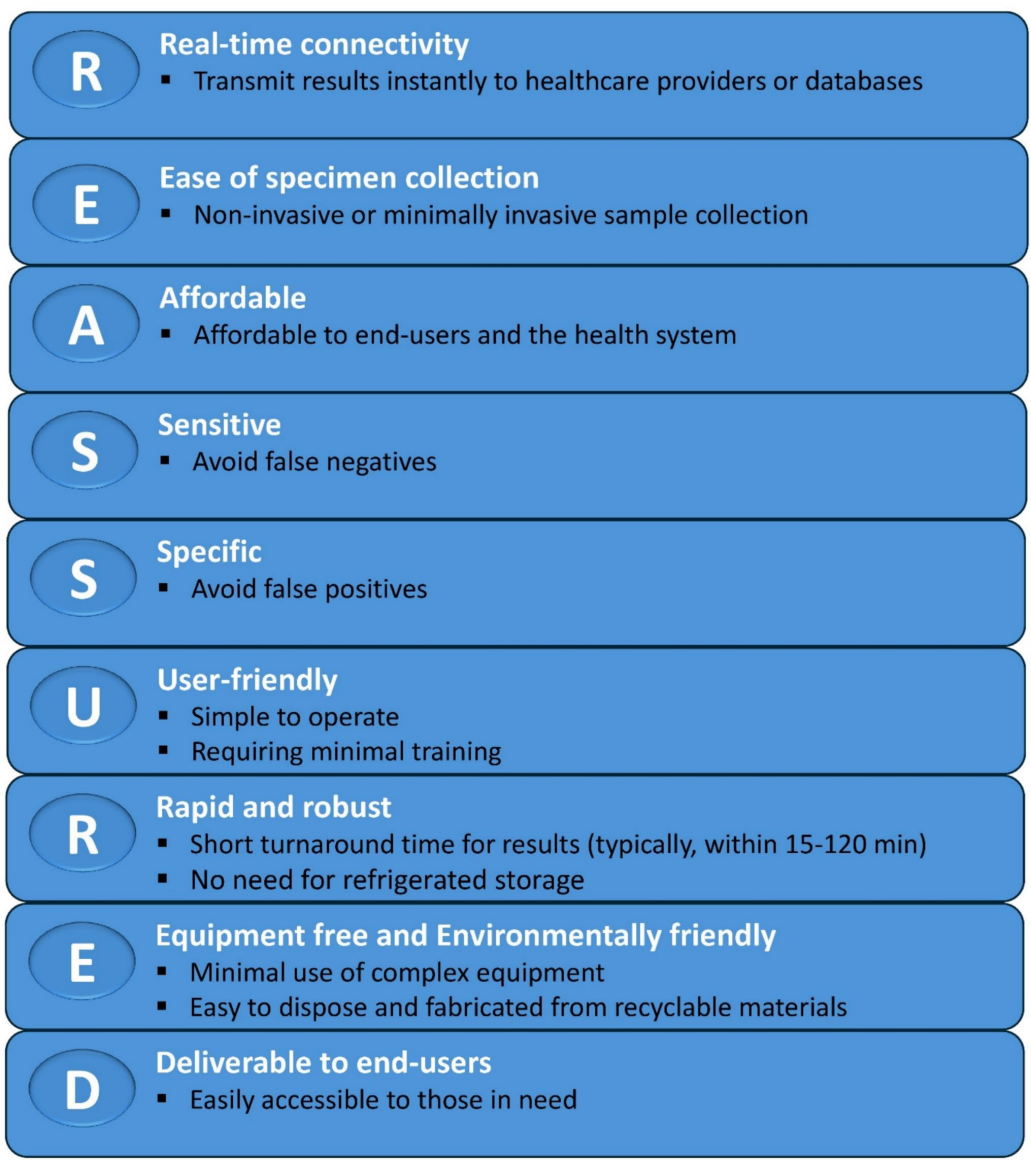
Requirements of an ideal POCT device suggested by WHO

POCT devices are a vital and transformational component of modern healthcare systems. In traditional diagnostic procedures, patients often need to visit healthcare centers, where samples are collected and sent to centralized laboratories for examination. This process can take days or even weeks to get results, thereby delaying timely decision-making and prompt patient care. Moreover, access to laboratory facilities is often limited for individuals in remote areas, creating challenges for proper diagnostics and medical care. However, POCT devices enable healthcare professionals and patients to quickly and conveniently access diagnostic information directly at the point of care, whether in a doctor’s office, a remote clinic, or even at home. In POC diagnostics, the analytical targets mainly include proteins, metabolites, nucleic acids, drugs, microorganisms, and human cells. The tests are typically performed on samples such as blood, urine, saliva, or other body fluids (Gubala et al. 2012). Interpretation of test results may be as simple as visually observing a color change on a test strip, or a clearer and more accurate result can be obtained using readers ranging from handheld instruments to benchtop devices. One of the earliest POCTs was reported by Kohn in 1957, who employed an enzyme-based test strip to estimate blood glucose levels (Kohn 1957). In the late 1970 s, the concept of at-home POCT emerged with the introduction of the first commercially available pregnancy tests (Ehrenkranz 2002). The personal glucose meter is one of the most widely commercialized POCT devices and has been available since the early 1980 s (Clarke and Foster 2012). In recent years, particularly during and after the COVID-19 pandemic, the demand for POCT has significantly accelerated (Rasmi et al. 2021; Kumar et al. 2022). Nowadays, this field is rapidly advancing through the development and integration of technologies such as lab-on-a-chip platforms, paper-based sensors, microfluidic, and wearable devices (Haghayegh et al. 2024; Li et al. 2024; Tandey et al. 2024; Rajarathinam et al. 2025). In 2003, WHO established a set of criteria known by the acronym ASSURED (“affordable, sensitive, specific, user-friendly, rapid and robust, equipment-free, delivered”), which has since become widely recognized as the benchmark for an ideal POC diagnostic test. Considering advances in emerging technologies, the ASSURED criteria were updated to include “real-time connectivity” (R), “ease of specimen collection, and environmental friendliness” (E), and are now known as the REASSURED criteria (Land et al. 2019) (Fig. 1).

## Biochemistry and clinical significance of troponins

**Fig. 2:**
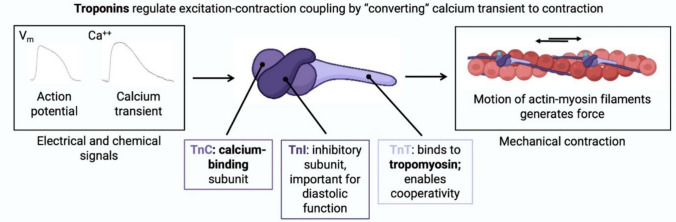
Structure and function of the troponin complex. Reproduced with permission from (Gokhan et al. 2024), copyright (2024), MDPI

The cardiac troponins are the most crucial regulatory proteins in the troponin-tropomyosin complex. This is found on the actin (thin) myofilaments in cardiac myocytes. It functions as a “switch” that permits the myosin motor proteins to attach to the actin filaments and start muscle contraction when calcium is present (Potter et al. 2022). The three subunits of this heterotrimer are troponin C (TnC), troponin I (TnI), and troponin T (TnT), each with distinct functions (Fig. 2) (Gokhan et al. 2024). cTnI is mainly found in the sarcomere and a small fraction in the cytosol. As an inhibitory subunit, the protein molecule cTnI prevents adenosine triphosphate from hydrolyzing and actin from interacting with myosin during the diastolic phase when calcium ions are not present. Two additional troponin subunits are attached to actin filaments by the protein molecule cTnT, which is a tropomyosin-binding subunit. As a calcium-binding subunit, the protein molecule cTnC binds calcium ions that enter the cytoplasm (Gokhan et al. 2024). Troponins are mostly found in the sarcomere, which is a component of the myofibrillar apparatus in cardiomyocytes. Only about 3.5% of total troponin I (TnI) and 7.0% of total troponin T (TnT) by mass are present in the cytosol (Chaulin 2021a). Studies have shown that the actual plasma half-life of cTnI and cTnT is comparatively short, at around 2 h (Sharma et al. 2004; Potter et al. 2022). However, because it reflects both continuous release from injured cardiomyocytes and removal from the circulation, the clinically observed half-life following myocardial infarction appears prolonged, normally 7 to 20 h (Kristensen et al. 2024). The elimination half-life for cTnI and cTnT in humans is approximately 2 to 4 h, according to recent research employing high-sensitivity assays and isolated elimination models (Potter et al. 2022; Kristensen et al. 2024). Greater peak troponin levels are linked to worse outcomes and greater mortality rates, and their size is correlated with the degree of heart injury. Troponin levels are helpful for both early and late detection of AMI, as they gradually decrease after peaking but can remain elevated for several days, up to 14 days for troponin T. However, serial measurements are necessary to record the distinctive rise and decrease in troponin concentration that characterizes AMI (Bhatt et al. 2016).

In addition to AMI, troponins may be elevated in a variety of cardiac and non-cardiac disorders. Heart failure, myocarditis, pericarditis, arrhythmias, cardiac contusions, and treatments such as cardiac surgery or percutaneous coronary intervention are examples of cardiac causes that can induce direct myocardial injury or elevated wall stress. Pulmonary embolism, chronic kidney disease, sepsis, stroke, critical illness, intense exercise, and even some surgical procedures are examples of non-cardiac causes, which are systemic diseases that result in myocardial strain, hypoxia, or impaired troponin clearance. The rise of troponin is hence not exclusive to acute coronary syndromes and must be interpreted in the clinical context, even if it is a sensitive indicator of myocardial damage (Jeremias and Gibson 2005; Korff et al. 2006; Zaki et al. 2022). The 99th percentile upper reference limit (URL) is used to determine the clinical cutoff for cTn assays. For example, the 99th percentile URL of cTnI, as measured using the ARCHITECT STAT (Abbott) laboratory assay and the PATHFAST hs-cTnI POCT device, are 0.0262 μg/L and 0.0290 μg/L, respectively. For cTnT, the value obtained using the Elecsys Troponin T Gen 5 STAT assay (Roche Diagnostics) is 0.0140 μg/L (Low et al. 2020). Below and above this value indicate normal and abnormal cTn conditions, respectively.

## Fundamentals of SERS and detection strategies

**Fig. 3:**
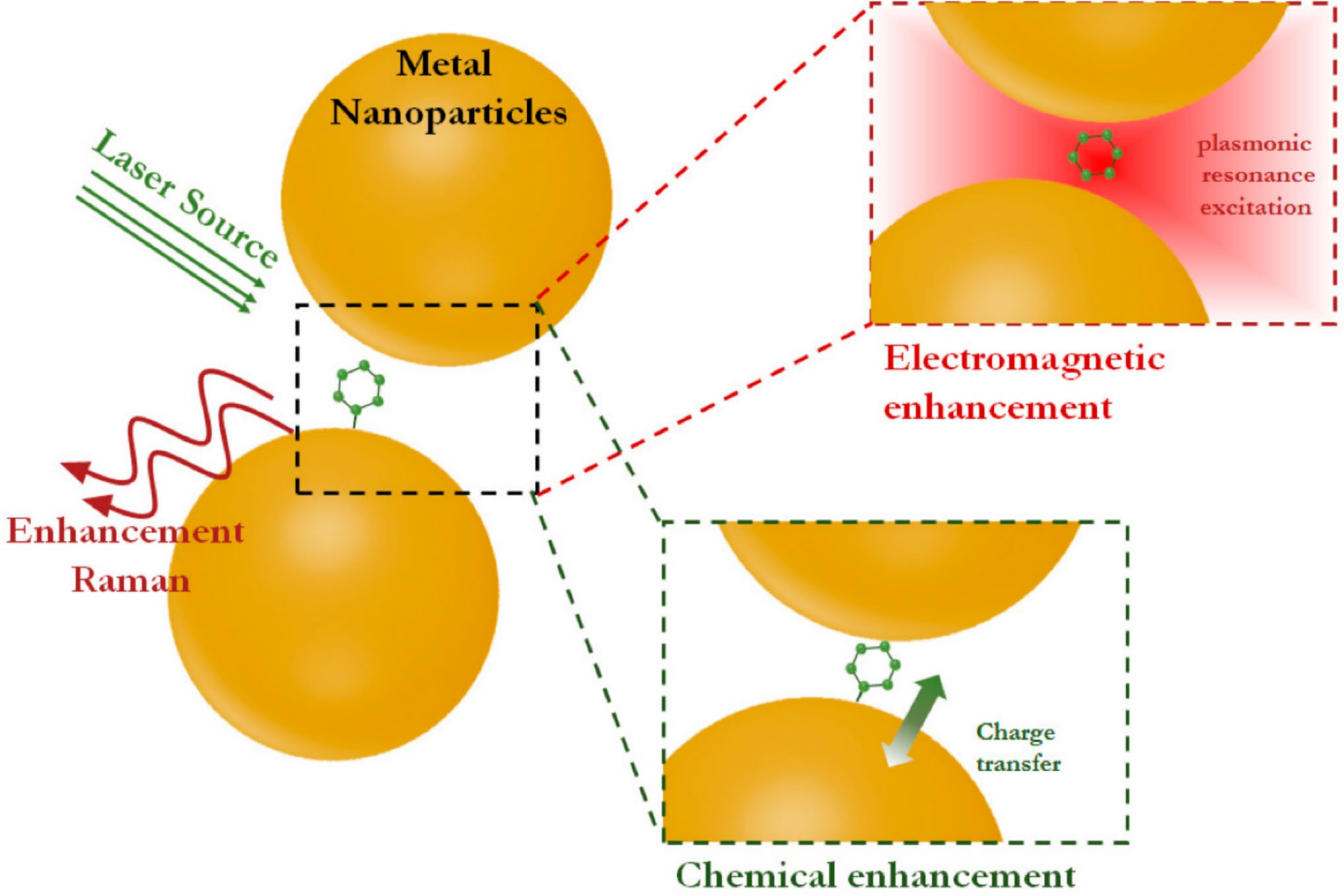
Illustration of the signal enhancement mechanisms in SERS. Reproduced with permission from (Serebrennikova et al. 2024), copyright (2024), MDPI

**Fig. 4:**
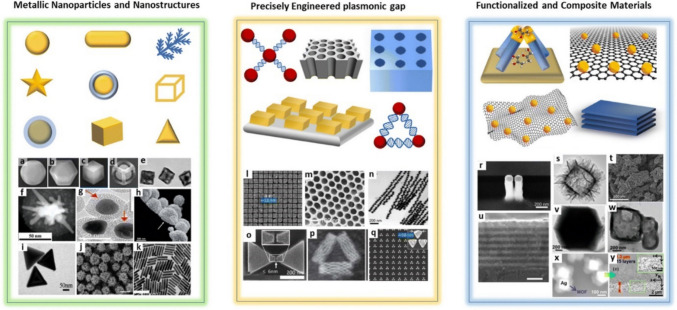
Schematic representations and corresponding SEM images of various types of plasmonic SERS substrates, (a) Au nanoparticle, (b) Au@Ag shell, (c) Au@Ag cage, (d) Au@Ag frame, (e) hollow Au nanocubes, (f) Au nanostar, (g) gap separated core–satellite nanoparticles, (h) mesoporous Au nanoparticle (i) Au nanotriangle (j) sea urchin-shaped Au particles (k) Au nanorods, (l) and (q) diverse Au structures with nanogaps, (m) porous Au substrate, (n) gap separated linear chain of Au nanoparticles, (o) bowtie nanoantenna, (p) triangular DNA origami (r) Au nanopillar on fused silica, (s) amorphous ZnO nanocage (t) semiconductor–metal hybrid (Au@DFH-4 T) nanostructure (u) atomic-layer WTe_2_ and MoTe_2_ substrate (v) metal–organic framework (ZIF-67), (w) porous metal–organic framework, (x) and (y) Ag with a metal–organic framework (Ag@MOF). Reproduced with permission from (Cialla-May et al. 2024), copyright (2024), The Royal Society of Chemistry

Raman spectroscopy is a powerful vibrational spectroscopic technique, often regarded as an ideal optical analytical tool, due to its ability to provide the unique molecular fingerprint of a sample based on vibrational modes specific to each molecule (Bankapur et al. 2010; Barkur et al. 2020; John et al. 2022a; Lukose et al. 2023). In this technique, monochromatic light ranging from near-infrared (NIR) to ultraviolet (UV) is employed to exploit the Raman effect, an inelastic scattering phenomenon that occurs when photons interact with molecules, leading to a shift in the energy of the scattered photons. Raman shift represents the difference in energy between the incident photon and the scattered photon caused by this vibrational transition. It is directly proportional to the frequency of the vibrational mode of the molecule that caused the scattering. Raman spectroscopy has proven effective in determining chemical components, examining molecular structures and conformations, and investigating interactions between molecules. Additionally, because the Raman signal of water is very weak, this technique can be easily applied to aqueous solutions without any complications. However, the application of the technique was limited due to the inherently weak Raman signal, which results from a lower Raman scattering cross-section compared to the fluorescence cross-section of the molecules. Additionally, in biological samples, the strong background autofluorescence often interferes, making it challenging to extract the Raman signals. These issues were successfully resolved in 1974 when Fleischmann and his team identified the surface-enhanced Raman scattering effect, observing significantly stronger Raman signals from pyridine adsorbed onto a roughened silver electrode (Fleischmann et al. 1974). In 1977, detailed independent studies by two groups revealed that the enhanced Raman signal originates from an increased scattering cross-section of adsorbed pyridine molecules (Albrecht and Creighton 1977; Jeanmaire and Van Duyne 1977). SERS has since become a crucial technique in bioanalysis, mainly due to its capability for ultrasensitive detection at the single-molecule level (Kneipp et al. 1997; Nie and Emory 1997) and its ability to enable multiplex detection with a single wavelength excitation (Zong et al. 2018). A critical aspect of the SERS technique is designing SERS substrates with various shapes, sizes, and coatings for diverse detection purposes. In particular, the SERS substrate can be designed for NIR lasers to minimize intrinsic autofluorescence from biological samples and reduce the photodamage to living cells caused by visible lasers (Zong et al. 2018). If the distance between the substrate and the target molecules is close (< 10 nm), the Raman signal can be enhanced by several orders of magnitude. It is now widely accepted that the electromagnetic (EM) and chemical enhancement mechanisms contribute to the overall signal enhancement in SERS effects (Chulhai et al. 2016). EM enhancement arises from surface plasmon resonances in plasmonic nanostructured substrates, which significantly amplify the local EM fields near their surfaces, boosting the Raman signals of nearby molecules by approximately 10^6^ to 10^8^ times. The enhancement factor is often well approximated by the fourth power of the magnitude of the localized EM field (Stiles et al. 2008). The EM field concentration occurs preferentially in nanoscale gaps, crevices, or sharp features of plasmonic materials, which are generally referred to as hot spots. In the chemical enhancement mechanism, the chemical interactions between the analyte molecule and the nanostructured substrate enhanced the Raman signal up to 10^2^–10^3^ times (López-Lorente 2021). The energy level structures of the target molecules and the substrate, and the excitation wavelength, are important in this mechanism (Morton and Jensen 2009). The schematic illustration of SERS signal enhancement mechanisms is shown in Fig. 3. From a materials perspective, noble metals are the most widely used for SERS substrates owing to their favorable plasmonic properties. Nanostructures made of gold (Au) and silver (Ag) exhibit excellent versatility and are easy to synthesize. They can be deposited onto solid supports through various bottom-up techniques to fabricate solid SERS substrates. Furthermore, the number of SERS hotspots can be increased by engineering the morphology of noble metal nanoparticles into structures such as nanostars, core–shell nanostructures, nanocubes, nanowires, nanovesicles, and nanofingers (Liu et al. 2013, 2018; Guo et al. 2015; Pan et al. 2019; Shao et al. 2019; Dong et al. 2022; Liu et al. 2023b; Ji et al. 2025). Au and Ag spherical nanoparticles have traditionally been the preferred choice for many SERS-based applications. However, anisotropic nanostructures with sharp edges or corners can generate stronger localized EM fields when illuminated by a laser. For example, a star-shaped Au nanoparticle substrate exhibits superior SERS performance for methylene blue dye with an enhancement factor of 10^8^, compared to solid spherical and hollow Au nanostructures, which show enhancement factors of around 10^6^ (Nehra et al. 2019). Compared to monometallic substrates, bimetallic SERS substrates such as Au@Ag and Ag@Au nanoparticles exhibit higher stability and significantly stronger SERS enhancement (Khaywah et al. 2015). Complex 3D plasmonic structures such as nanoframes, nanocages, and bimetallic nanostructures can enhance signal intensity by effectively trapping analyte molecules and utilizing the combined properties of multiple metals (Mitra and Basak 2022). A metallic array substrate in SERS typically consists of highly ordered nanoscale metallic structures, such as pyramidal or nanosphere arrays, that create hot spots for greatly amplifying Raman signals (Zhang et al. 2018; Wu et al. 2021). In 2010, the two-dimensional material graphene was explored for SERS applications (Ling et al. 2010). Other two-dimensional layered materials used in SERS include molybdenum disulfide (MoS₂) and hexagonal boron nitride (h-BN) (Ling et al. 2014). In such materials, Raman signal enhancement arises from the chemical mechanism, which involves charge transfer between the material and the target molecules. Semiconducting materials such as ZnO and TiO_2_ are used as SERS substrates, offering greater possibilities for modulating SERS activity through a chemical enhancement mechanism (Wang and Guo 2020). An illustrative summary of different types of plasmonic SERS substrates is shown in Fig. 4.

**Fig. 5:**
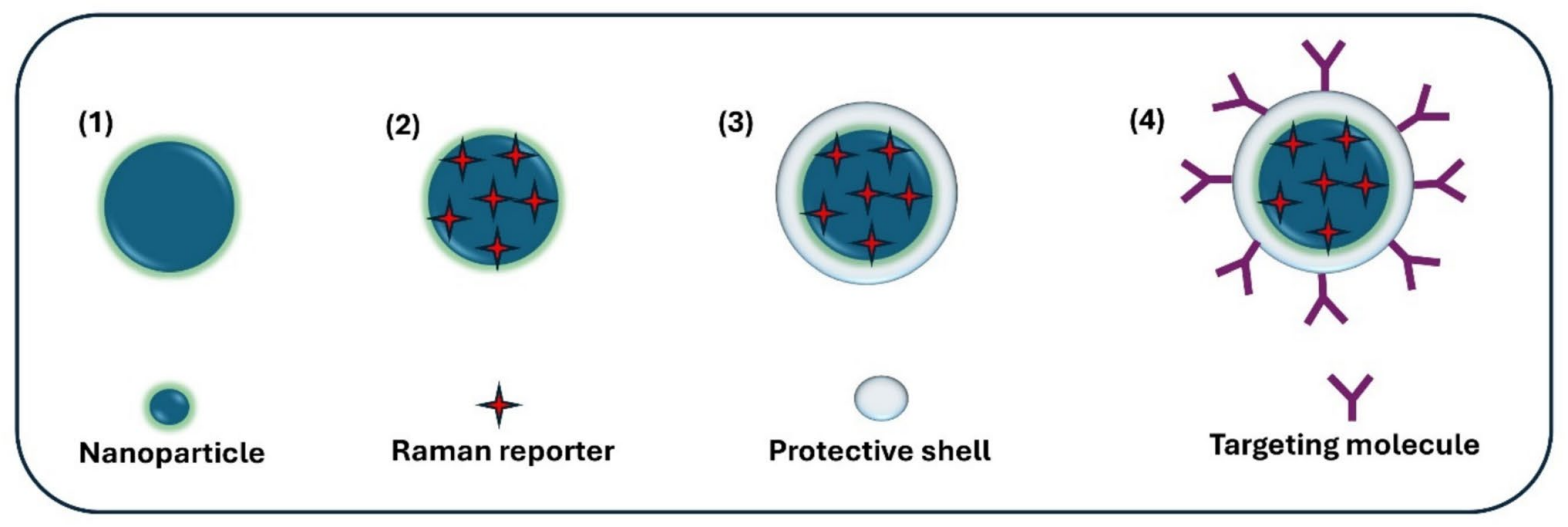
Fabrication procedures of SERS nanotag. (1) Design of SERS substrate. (2) Functionalization with Raman reporter. (3) Protective shell coating. (4) Biofunctionality

SERS enables the detection and quantification of analytes through two main approaches: direct and indirect. Direct detection, also known as “label-free” detection, does not require any additional labels (Barkur and Chidangil 2018). In this approach, the target molecules interact directly with the nanostructured SERS substrate, and the enhanced Raman signal originates directly from the target molecules themselves. In contrast, indirect detection focuses on monitoring the spectral bands of a distinct, well-characterized molecule commonly called a “reporter” or “label” that is linked to the target molecules. The Raman bands of these reporters can appear, disappear, or shift upon interaction with the target molecules (Cialla-May et al. 2024). Indirect detection is the preferred approach when the target analyte has a low Raman cross-section, is not sufficiently close to the nanostructured metallic substrate to benefit from the enhanced electromagnetic field, exhibits competing phenomena such as fluorescence, or when the resulting SERS signal fails to meet the required analytical standards. The indirect approach helps manage the inherent complexity of SERS by employing Raman reporter molecules (RRM) with strong and predictable SERS spectra, providing researchers with greater control over the system. However, this control comes with a trade-off: the reporter must be linked to the target analyte through a direct covalent bond or via a recognition element such as an aptamer or antibody (Cialla-May et al. 2024). In the indirect detection method, the terms SERS tag and SERS probe refer to a core nanoparticle (NP) or a cluster of NPs coated with RRM and functionalized with a target-specific ligand (Oliveira et al. 2022). A biocompatible shell can be introduced between the RRM and the recognition biomolecule to retain the RR close to the NP, thereby maximizing SERS signal enhancement and stabilizing the NPs. Each component of the SERS tag, such as the type of NP (typically Au or Ag), its size and shape, the NP configuration (single or clustered), the choice of RRM, the protective shell type, and the type of bioconjugation process, can be tailored based on the specific requirements of the intended application. When selecting RRM, several key factors must be considered. Firstly, the reporter should exhibit a narrow frequency distribution, easily recognizable Raman spectral features, and a large Raman scattering cross-section. RRM should be readily and firmly attached to the surfaces of plasmonic nanoparticles. To ensure reliable detection in biological samples, reporter molecules should exhibit high resistance to photobleaching. Additionally, according to the chemical enhancement mechanism of SERS, RRM must strongly interact with the substrate and form chemical bonds to generate molecular hotspots that significantly amplify the Raman signal. Smaller molecules are generally more suitable as reporters than larger ones, due to their shorter interaction distances and stronger binding affinity to the substrate. For bioanalysis, commonly used reporter molecules include dye compounds such as rhodamine 6G (R6G), indocyanine green (ICG), and crystal violet (CV), as well as molecules that form strong bonds with the substrate and contain bonds like N − H or S − H, such as 4-mercaptobenzoic acid (4-MBA), 4-aminothiophenol (4-ATP), 4-mercaptopyridine (4-MPY), and p-aminothiophenol (P-ATP) (Lian et al. 2025). The fabrication procedures of the SERS nanotag are shown in Fig. 5.

**Fig. 6:**
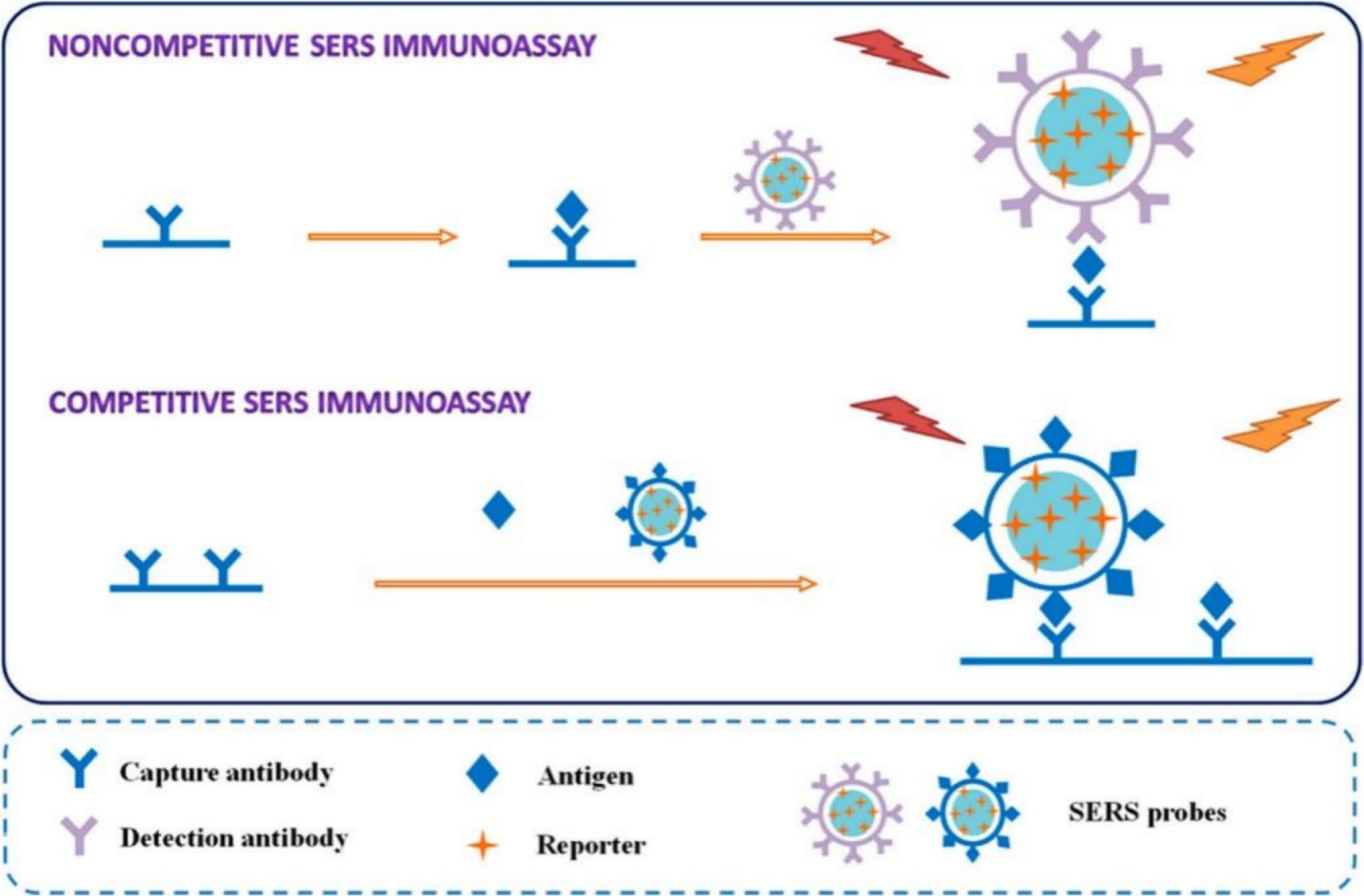
Mechanism of non-competitive and competitive SERS immunoassay. Reproduced with permission from (Wang et al. 2017b), copyright (2017), American Chemical Society

An immunoassay is one of the most widely used analytical techniques, relying on the specific interaction between an antigen and a complementary antibody for the quantitative detection of biochemical molecules such as proteins, hormones, and biomarkers (Wu et al. 2012). A key feature of all immunoassays is their ability to generate a measurable signal upon an analyte binding. SERS-based immunoassays are those that utilize surface-enhanced Raman scattering as the detection or readout signal. The first standard SERS-based immunoassay, employing a sandwich format, was developed by Ni et al. in 1999 (Ni et al. 1999). Over the following decades, SERS-based immunoassay platforms have rapidly evolved from metallic (Ni et al. 1999) and non-metallic (Li et al. 2012) solid substrates to magnetic beads in aqueous phase (Zong et al. 2013) and further to innovative formats such as paper-based devices (Su et al. 2021) and microfluidic chips (Gao et al. 2019). Typically, a SERS immunoassay comprises two main components: the immunoprobe and the immune substrate. To capture a specific analyte molecule from the sample, the immune substrate is usually functionalized with targeting molecules. SERS probes have two basic roles: first, to specifically recognize and bind to analyte molecules captured by the immune substrate; second, to generate SERS signals for their quantitative detection. Based on the binding mechanism of the immunocomplex, SERS-based immunoassays can be categorized as either competitive or noncompetitive, the latter also referred to as “sandwich” or “two-site” immunoassays (Fig. 6) (Wang et al. 2017b). As the name suggests, typical sandwich immunoassays employ a sandwich format, where the target molecules are sandwiched between the SERS probes and the capture antibodies. These types of immunoassays are suitable for analyzing macromolecules such as proteins. In noncompetitive SERS immunoassays, the intensity of the SERS readout signal is positively correlated with the amount of target present: higher target concentrations result in the formation of more sandwich complexes, leading to a stronger SERS signal. In competitive immunoassays, the target molecules compete with SERS probes for binding to the antibodies and are typically used to detect small molecules. In this case, the intensity of the measured signal is inversely correlated with the target concentration.

The aptamer-based assay is a promising alternative to traditional immunoassays and is receiving growing attention in the bioanalytical community. Aptamers, also known as “chemical antibodies,” are short, single-stranded oligonucleotides (or peptides) that specifically bind to target molecules through their unique three-dimensional conformation (Arshavsky‐Graham et al. 2022) . Aptamer–target binding is noncovalent and mediated by forces such as van der Waals interactions and hydrogen bonds, forming a three-dimensional conformation such as a stem, hairpin, loop, or G-quadruplex (Patel 1997). One of the earliest attempts at SERS-based aptamer assays was reported by Wang et al. in 2007, where they developed a simple SERS aptasensor for protein detection using gold nanoparticles (AuNPs) functionalized with aptamers and Raman reporters with a detection limit of 0.5 nM (Wang et al. 2007). Aptamers possess numerous advantages, such as simple and cost-effective synthesis, minimal immunogenicity, high stability under varying pH and temperature conditions, and superior long-term storage stability compared to conventional recognition elements like antibodies (Lee et al. 2008; Ilyas et al. 2023).

SERS measurements can be conducted in platforms such as solid substrates (e.g., glass or metal surfaces) and magnetic beads in aqueous suspension will hereafter be referred to as traditional SERS platforms, to distinguish them from comparatively new approaches such as microfluidic- and paper-based platforms.

## Traditional SERS platforms

**Fig. 7:**
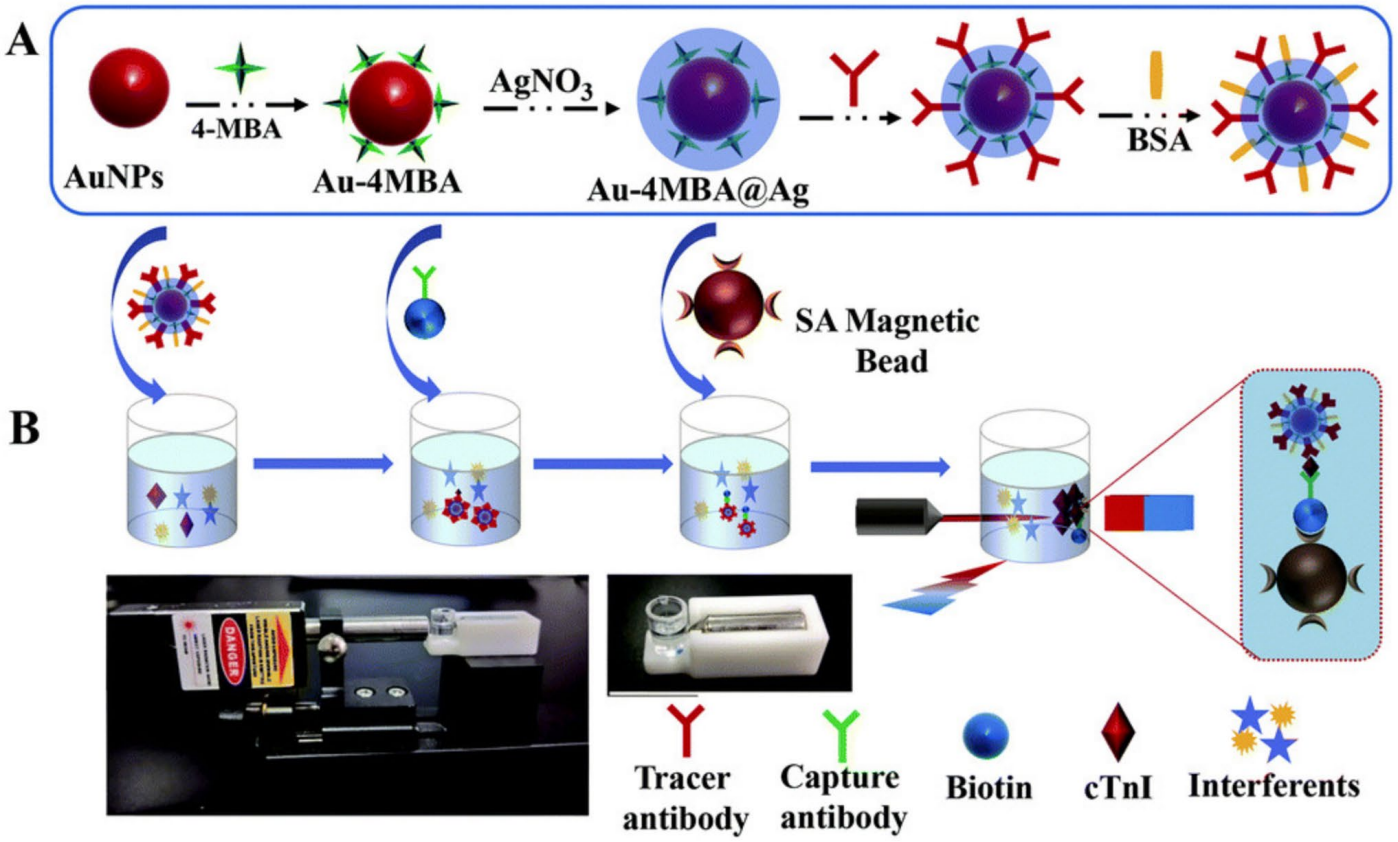
**A** Core–shell (Au-4MBA@Ag) SERS immunoprobe preparation process and **B** schematic of cTnI detection by SERS-based sandwich immunoassay, the actual detection picture is also inserted. Reproduced with permission from (Hu et al. 2021), copyright (2021), The Royal Society of Chemistry

Most SERS-based immunoassays and apta assays for cTn detection utilize a sandwich format. Fu et al. reported a SERS-based sandwich immunoassay for detecting cTnI (Fu et al. 2019). In this work, AuNPs modified with antibodies and the Raman reporter malachite green isothiocyanate were further conjugated with graphene oxide (GO) to form the SERS nanotags. Magnetic beads functionalized with antibodies served as the capture substrates. Rabbit polyclonal antibodies to cTnI were used as the detection antibodies, while mouse monoclonal anti-cTnI antibodies were used to functionalize the magnetic beads. Capture substrate/cTnI/SERS nanotag sandwich immunocomplexes were formed by first adding cTnI to the capture substrate suspension, followed by incubation. The complexes were then separated using an external magnet and washed with PBS. Subsequently, SERS nanotags were added and incubated. The immunocomplexes were isolated, washed, and redispersed in PBS buffer. The resulting supernatant was then transferred into a capillary tube for SERS measurements. The proposed immunoassay demonstrated a LOD of 5pg/mL, whereas without the use of GO, quantification of cTnI was challenging at concentrations below 1 ng/mL. A SERS-based sandwich immunoassay platform was developed for the detection of cardiac biomarkers cTnI and CK-MB (Cheng et al. 2019). To enable target antigen capture, monoclonal antibodies specific to cTnI and CK-MB were immobilized onto a gold-patterned chip. The chip was fabricated by depositing AuNPs onto the surface of the silicon wafers using the immersion method, followed by transferring and assembling the wafers onto a glass slide. Subsequently, Au@Ag core–shell, conjugated with corresponding polyclonal antibodies, were introduced to form sandwich-type immunocomplexes. The SERS probes were labelled with the RRM MGITC, and the characteristic Raman signal at 1615 cm⁻^1^ representing the strongest intensity was used for quantitative analysis. The LODs for cTnI and CK-MB estimated by this assay were 8.9 pg/mL and 9.7 pg/mL, respectively, demonstrating a sensitivity approximately 2 or 3 orders of magnitude greater than that of conventional immunoassay techniques such as fluorescence-based assays or ELISA. For the simultaneous detection of cTnI and heart-type fatty acid-binding protein (H-FABP), a SERS-based sandwich immunoassay was developed by Hu et al. (Hu et al. 2020). Two RRMs, 4MP and XP013, were embedded in the gap between the Au core and Ag shell nanoparticles to form Au-RRMs@Ag core–shell SERS nanotags. These nanotags were then conjugated with tracer antibodies to form SERS immunoprobes, while biotin modified with capture antibodies was used to form the capture probe. In the presence of target biomarkers, SERS nanoprobes/target biomarker/capture probe sandwich complexes were formed through an immune reaction. These immunocomplexes were subsequently separated using streptavidin-modified magnetic beads, and Raman measurements were then performed. The biomarker concentrations were quantified by measuring the characteristic Raman peak intensities of 4MP at 1073.5 cm⁻^1^ and XP013 at 1377.3 cm⁻^1^. The cTnI values measured in clinical serum samples using the proposed assay showed good correlation with those obtained by the chemiluminescence method. The same research group in 2021 reported a similar strategy of SERS-based sandwich immunoassay for cTnI detection, utilizing Au-4MBA@Ag core–shell nanotags (Hu et al. 2021). Figure 7 illustrates the preparation of the SERS immunoprobes, and the overall detection strategy employed for cTnI using this immunoassay approach. In this work, the entire analysis procedure was performed in a test tube and completed in approximately 30 min using a portable Raman device, establishing a basis for POCT. Wang et al. developed a SERS immunoprobe by embedding 4-MBA within the core–shell gap of Au@Ag nanospheres and functionalizing the surface with antibodies (Wang et al. 2022). Bovine serum albumin (BSA) was used as a protective layer to enhance the stability of the nanospheres. Compared to the methods by Hu et al. (Hu et al. 2020, 2021), the absence of magnetic beads simplified the synthesis of the immunocomplex, and the analysis could be completed within 15 min, making this assay a tool for the rapid detection of cTnI. A microcavity-based immunochip in a sandwich format for cTnI detection via SERS was developed, in which the capture chip consisted of polystyrene microspheres modified with Au NPs immobilized on a substrate using polydopamine, and DTNB-labelled Au NPs served as immune probes (Wang et al. 2021). The calculated SERS enhancement factor value for the capture chip was up to 1.95 × 10^12^. In whole blood samples, the recovery rates of cTnI ranged from 109.4% to 121.6%, and the average coefficient of variation between replicates was below 13%. Recently, Xiang et al. developed a SERS-based sandwich immunoassay for detecting cTnI, employing ordered arrays of multi-tipped Au nanostars on silicon wafers as the SERS substrate (Xiang et al. 2025). These nanostar arrays exhibited approximately a threefold increase in Raman signal enhancement, as well as improved uniformity and stability, compared to conventional Au nanosphere-based substrates. The primary antibody, cTnI-McAb-29, was immobilized on the SERS substrate using 11-mercaptoundecanoic acid as the linker. 4-MBA was employed as the RRM to label Au NPs, which were further functionalized with the cTnI-McAb-30 antibody to serve as the SERS probe. The antigen proteins (cTnI-Ag5) and the SERS immunoprobe were sequentially incubated on the immune substrate, resulting in the formation of a sandwich immunocomplex through specific binding between the antigen and antibody. The proposed SERS detection strategy, relying on plasmonic enhancement from both the SERS immunoprobe and immune substrate, enables a LOD as low as 9.09 pg/mL. This immunoassay exhibits reproducibility, with an RSD value of 11.24%. Lee et al. demonstrated the feasibility of integrating a portable Raman device with a SERS-based immunochip for simultaneous and individual quantitative detection of cTnI and CK-MB (Lee et al. 2024a). In their approach, 2-NAT and 4-MBA RRMs were conjugated to Au nanocubes (AuNCs) to serve as SERS tags for labelling cTnI and CK-MB, respectively. These nano tags were further functionalized with detection antibodies to form immuno probes. In the presence of target proteins, these probes bind to capture antibody-conjugated Au substrates, forming a sandwich immunocomplex for sensitive and specific detection. The authors developed a rapid immunoassay protocol that enables the reaction between nanoprobes and serum to be completed within 10 min (conventional protocols, which typically take over 2 h), further demonstrating the potential for POCT applications. Their findings showed that the SERS immuno-chips, when used with both benchtop and portable Raman devices, accurately detected cTnI and CK-MB biomarkers after the onset of AMI. SERS substrates are typically fabricated by depositing plasmonic NPs onto bulk materials such as glass, silicon, or metal wafers (Xu et al. 2019). Recently, Ma et al. reported an in situ-synthesized Au–Ag NPs-decorated cellulose paper as a SERS substrate for cTnI detection (Ma et al. 2025). The cellulose paper substrate generates minimal background, and interference signals and offers advantages such as flexibility, biocompatibility, biodegradability, and low cost. The PEG1 (HS-PEG-COOH) linker was used to covalently bind the capture antibody, forming the SERS immune substrate. The reporter DTNB-modified Au core is coated with an Ag shell to form Au@DTNB@Ag, which is then functionalized with an antibody to form a SERS immunoprobe. Upon interaction with the immune substrate in the presence of cTnI, a sandwich immunocomplex is formed. The quantification of the biomarker is achieved by measuring the intensity of the characteristic Raman peak at 1332 cm^−1^. This assay achieved a short detection time of 10 min and a LOD of 10.79 fg/mL for cTnI.

**Fig. 8:**
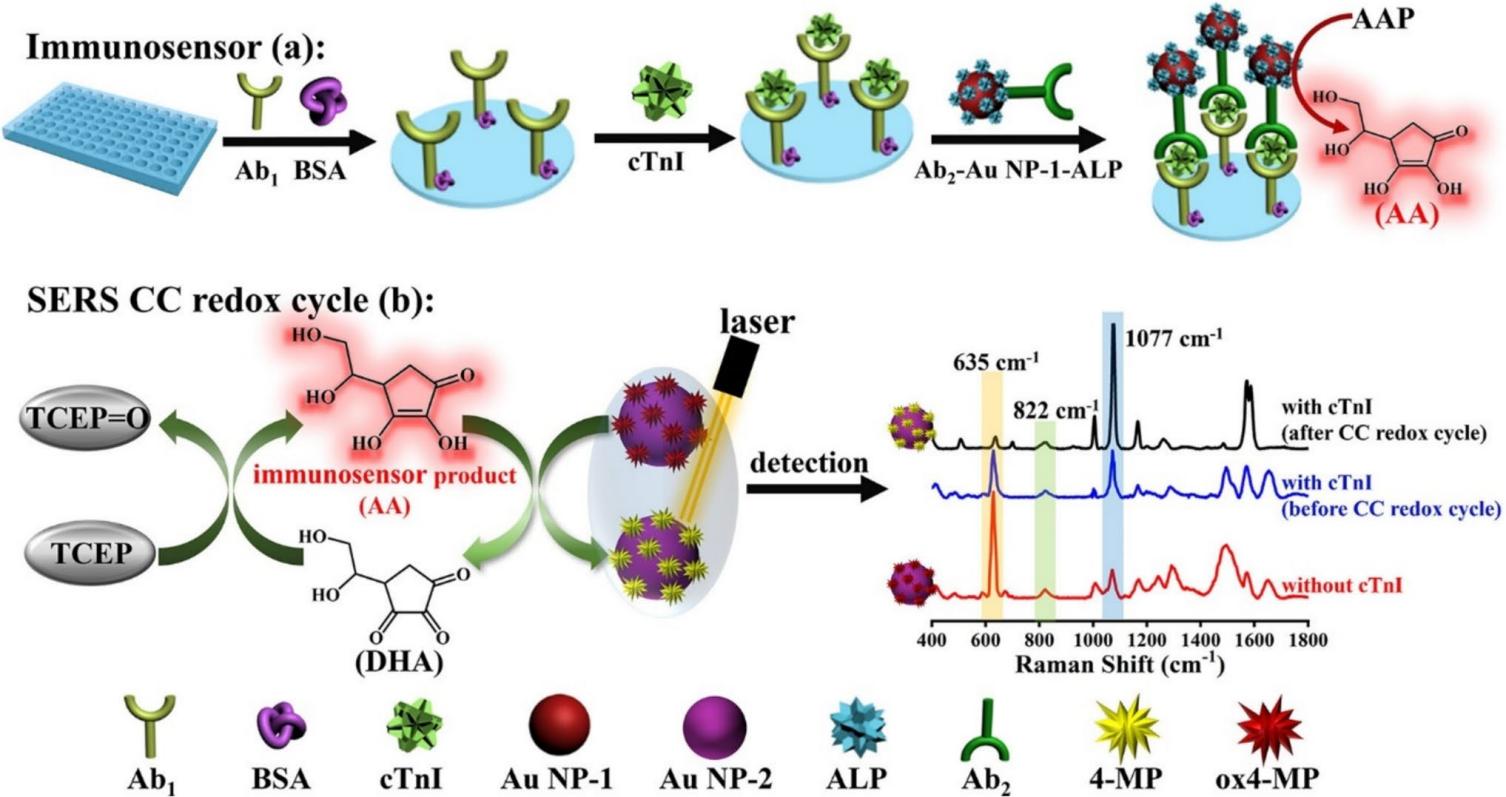
Schematic diagram of cTnI detection based on chemical–chemical (CC) redox cycle amplification combined with ratiometric immunoassay strategy. Reproduced with permission from (Zhao et al. 2023), copyright (2023), American Chemical Society

SERS signal amplification via a chemical–chemical redox cycle, integrated with a dual ratiometric immunoassay for cTnI detection, was introduced by Zhao et al. (Zhao et al. 2023). For the construction of the immunoassay, antibody (Ab1) specific for cTnI and BSA were immobilized onto the 96-well plate. Following the addition and incubation of cTnI, Au NPs-1 modified with alkaline phosphatase (ALP) and antibody (Ab2) specific for cTnI were sequentially added to form a sandwich immunocomplex. This enzyme-assisted immunoreaction catalyzes the hydrolysis of ascorbic acid 2-phosphate (AAP) to produce ascorbic acid (AA). Then, oxidized 4-MP (ox4-MP) attached to Au NPs-2 reacted with AA to generate 4-MP and dehydroascorbic acid (DHA). Subsequently, in the presence of tris(2-carboxyethyl) phosphine (TCEP), DHA was reduced, regenerating AA (Fig. 8). As the concentration of cTnI increases, there is a gradual increase in the peak at 1077 cm^−1^ (while the peak at 635 cm^−1^ reduces) due to the increased amount of AA generated by the immunoreaction, which in turn raises the 4-MP content. The peak at 822 cm^−1^ remains unaltered during the detection. In this assay, the intensity ratios of SERS signals I_1077_/I_822_ and I_635_/I_822_ are used for the quantification of cTnI. Most immunoassays for cTnI detection employ a sandwich format; recently, Yoo et al. employed a competitive immunoassay for its detection (Yoo et al. 2025). In their SERS substrate fabrication, heat treatment (up to 800 °C) was used to create protruding grain structures on the surface of the nickel foam, followed by Au deposition that produced densely packed and uniform hemispherical structures approximately 20 nm in diameter on the grains. The SERS substrate exhibited reusability, which was demonstrated through multiple cleaning and detection cycles. The reproducibility of the assay was demonstrated by a relative standard deviation (RSD) of 4.6%. In another study, instead of a noble metal SERS substrate, perovskite quantum dots encapsulated in a metal–organic framework and combined with graphene nanosheets (CsPbBr_3_@ZIF-8@G) were fabricated as a SERS substrate for cTnI detection (Qin et al. 2024). The use of graphene can suppress the fluorescence of CsPbBr_3_@ZIF-8. In this study, carboxy-functionalized magnetic beads conjugated with cTnI antibodies are used as capture probes. The magnetic bead–antibody complex is mixed with the sample containing cTnI and incubated to allow binding. After isolating the bead–antibody–cTnI complex via magnetic separation, the complex is stained with Coomassie bright blue G250 (CBBG). The stained complex is magnetically separated, and the supernatant containing unbound CBBG is collected. The supernatant is then dropped onto the SERS substrate, and Raman measurements are conducted. The reverse SERS method is employed, which indirectly quantifies cTnI by measuring the decrease in the signal from CBBG as the cTnI concentration increases. This method achieved recovery rates in human serum samples ranging from 93.1% to 104.8%, with RSD values between 4.47% and 7.06%.

Alves et al. developed a label-free SERS-based aptasensor for the detection of cTnI, utilizing flower-shaped Fe_3_O_4_@SiO_2_@Ag nanoparticles functionalized with a 5’-thiol-modified aptamer as the capture probe (Alves et al. 2020). For the SERS measurements, as-prepared capture probe suspension containing cTnI was directly dropped onto a silicon substrate. The sample was concentrated by placing a cylindrical Nd magnet beneath the substrate, then dried under magnetic attraction, and the SERS spectra were subsequently recorded. Concentrations of cTnI as low as 10 ng/mL could be detected using this SERS aptasensor. Lin et al. synthesized a SERS capture probe consisting of a bimetallic magnetic substrate, Fe_3_O_4_@Ag@Au, functionalized with a sulfhydryl-modified aptamer via an Au–S bond (Lin et al. 2022b). The magnetic capture probe can selectively bind cTnI from the sample through the specific interaction between the aptamer and the target protein, after which the complex binds to Coomassie Brilliant Blue G-250 (CBBG), the Raman signal molecule for quantitative measurement. Their detection strategy involves adding serum samples (without any pretreatment or dilution) to an EP tube containing a magnetic capture probe, followed by ultrasonic incubation. After removing unbound substances with PBS through magnetic separation, CBBG is added and allowed to react for 10 min at room temperature. Once the Raman signal molecule–cTnI–magnetic capture probe complexes were formed, they were collected via magnetic separation, washed with PBS, and subsequently redispersed in PBS. Finally, the redispersed solution was applied onto the surface of a glass slide placed on a magnet, and the Raman signals were recorded while drying. When the storage time of the analyte complex with CBBG exceeds 5 days, the Raman measurement results show a significant deviation from the actual cTnI concentration. To verify the accuracy of the detection method in real serum samples, a recovery test using the standard addition method was performed by spiking healthy human serum with varying concentrations of the target cTnI. The recovery rates and RSD of this experiment ranged from 92 to 115% and 7.4% to 12.7%, respectively. Lee et al. reported an aptamer-based SERS sandwich assay for the detection of cTnI (Lee et al. 2020). A probe aptamer with a thiol group was immobilized on an atomically flat gold nanoplate platform, serving as the capture substrate. The reporter aptamer, with Cy5 (a Raman dye) and a thiol group, was attached to gold nanoparticles to form the detection probe. Using this SERS method, cTnI could be detected at concentrations of 2.4 fg/mL in buffer solution and 2.4 pg/mL in serum. A novel SERS method for cTnI detection, based on the specific aptamer–target protein interaction and the bicinchoninic acid (BCA) method, was proposed by Lin et al. (Lin et al. 2022a). In this study, aptamers were functionalized on the surface of coral-like nano-silver-modified magnetic particles via Ag–S bonds to form the magnetic capture probe (Fe₃O₄@PEI/Ag NC-Apt). The magnetic capture probe can specifically recognize and isolate cTnI in serum samples through aptamer–target interactions. The captured complex then reacts with the BCA working solution, followed by Raman measurement performed on a glass slide placed over a magnet, utilizing the Raman enhancement effect of the coral-like nano-silver structure in the capture probe. The spiked tests conducted on human serum samples yielded recovery rates between 92 and 106%, and RSD ranging from 3.8% to 10.1%. In a recent study, Wang et al. proposed a SERS-based ratiometric aptasensor for quantifying cTnI in human serum samples, in which the capture substrate and SERS probes form a sandwich structure (Wang et al. 2024b). The aptamer Tro4 (modified with sulfhydryl groups) was immobilized on a uniform, high-density gold array, which acted as the capture substrate. To calibrate signal fluctuations, 5,5’-dithiobis-(2-nitrobenzoic acid) (DTNB) was anchored to the gold array via Au–S bonds. SERS probes were synthesized by functionalizing porous gold (pAu) with the aptamer Tro6 and 4-mercaptobenzoic acid (4-MBA). The ratiometric aptasensor functions such that, with increasing concentrations of cTnI, a greater number of SERS probes are bound in a sandwich format to the capture substrate, resulting in an enhanced 4-MBA signal at 1585 cm^−1^, while the DTNB signal at 1331 cm^−1^ remains unchanged. The ratio of SERS signal intensities at 1585 cm^−1^ to 1331 cm^−1^ was used to quantify the concentration of cTnI. A recent study by Wu et al. presented another ratiometric SERS-based assay for the detection of cTnI, sST2, and NT-proBNP (Wu et al. 2025). In this study, Au@Ag NPs functionalized with the RRM 2-mercaptopyridine (2-MPY) and specific aptamers/antibodies for cTnI serve as SERS probes. Magnetic Fe_3_O_4_ NPs coated with an Ag shell and modified with the internal standard molecule 4-mercaptobenzonitrile (4-MBN), which has a peak at 2224 cm^−1^, act as the capture substrate. SERS probe–cTnI–capture substrate sandwich complexes are formed, which are then magnetically isolated and concentrated using an external magnetic field for subsequent SERS measurement in a solid substrate. The reproducibility test was conducted in a mixed solution containing all three biomarkers, and the RSD for cTnI was calculated to be 9.24%. The recovery rates for cTnI in PBS buffer ranged from 96.00% to 106.28% at spiked concentrations from 0.5 to 50.0 pg/mL. The intra-batch CV of each biomarker ranged from 5.87% to 9.06%, and the inter-batch CV for different SERS substrates was between 9.12% and 10.10%. For clinical validation, the SERS technique was compared with two clinical platforms—the Roche Cobas 8000 e602 and the immune F6 automatic chemiluminescence immunoassay analyzers and showed good agreement with both.

## Microfluidic and paper-based platforms

According to the definition of George M. Whitesides, microfluidics is “the science and technology of systems that process or manipulate small (10^–9^ to 10^–18^ L) amounts of fluids, using channels with dimensions of tens to hundreds of micrometres” (Whitesides 2006). A microfluidic chip, also referred to as Lab-on-a-Chip, is a compact device that integrates multiple laboratory functions onto a single platform (Sackmann et al. 2014; Ho et al. 2015). Microfluidic chips typically consist of sample inlets and outlets, microchannels, micropumps, microchambers, and microvalves. They can perform automated and highly accurate biological and chemical processes, with sample mixing, reaction, isolation, and detection all occurring on a single chip. Microfluidic chips offer unique advantages, including low sample consumption, high throughput, rapid reaction times, efficient mass transfer, and high customizability (Lian et al. 2025). Fluid transport in microfluidic systems can be achieved either in a passive mode (without power sources), utilizing gravity and capillary forces, or in an active mode, employing external power sources or actuators (Kant and Abalde-Cela 2018; Narayanamurthy et al. 2020).

SERS-based microfluidics combines microfluidic technology with SERS detection, has attracted considerable interest from researchers due to its strong potential in POCT applications (Lee et al. 2024b). The accurate quantification of biomolecules using SERS remains challenging due to limitations in signal reproducibility. However, sensitive and reproducible SERS detection is accomplished by leveraging the average ensemble effects of nanotags rapidly moving through microfluidic channels (Park et al. 2022; Lee et al. 2024b). For SERS applications, microfluidic networks create a continuous flow environment that ensures uniform mixing, supports heat dissipation, and minimizes nonspecific binding of SERS tags. This helps reduce the variability typically observed in SERS assays, which arises from a lack of control over the aggregation of NPs, size, and distribution of analyte on the detection surface (Langer et al. 2020; Oliveira et al. 2022).

**Fig. 9:**
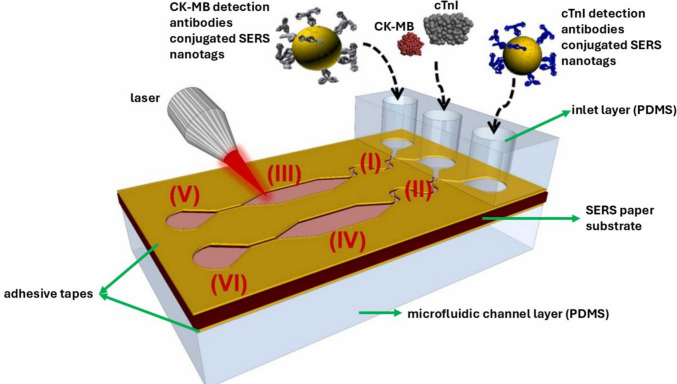
Schematic representation of hybrid microfluidic chip with pump-free design for simultaneous SERS detection of dual cardiac biomarkers. Adapted with permission from (Gao et al. 2022a), copyright (2022), Elsevier

Gao et al. designed a pump-free microfluidic chip composed of a PDMS microchannel hybridized with a microchannel-patterned filter paper SERS substrate, where AuNPs were in-situ synthesized throughout the entire paper channels (Gao et al. 2022a). In combination with the PDMS microchannel, the continuous flow of aqueous samples through the paper microchannels was driven by the capillary action of the cellulose fibers. The SERS-based microfluidic chip for simultaneous detection of cTnI and CK-MB consists of five layers, as illustrated in Fig. 9: two PDMS layers (inlet layer and microfluidic channel), two adhesive double-sided tapes and a paper SERS substrate. Two double-sided adhesive tapes with hollow microchannel design were used to integrate the two PDMS layers and the paper SERS substrate into a single hybrid microfluidic chip (39 mm × 26 mm). The SERS nanoprobes (MGITC and detection antibodies for cTnI/CK-MB conjugated to AuNPs) and the serum spiked with the two corresponding antigens were introduced into three separate inlets using a multichannel pipette. The mixing of samples and the immuno-reaction take place within the 500 μm-wide winding channels (I and II in Fig. 9). “Sandwich” immunocomplexes are formed in the hexagonal chamber (12 mm in length and 4 mm in width), which is pre-immobilized with capture antibodies (III and IV). Finally, the unbound components are washed away by adding PBS buffer solution, which then flows to the outlets (V and VI), followed by SERS measurements.

**Fig. 10:**
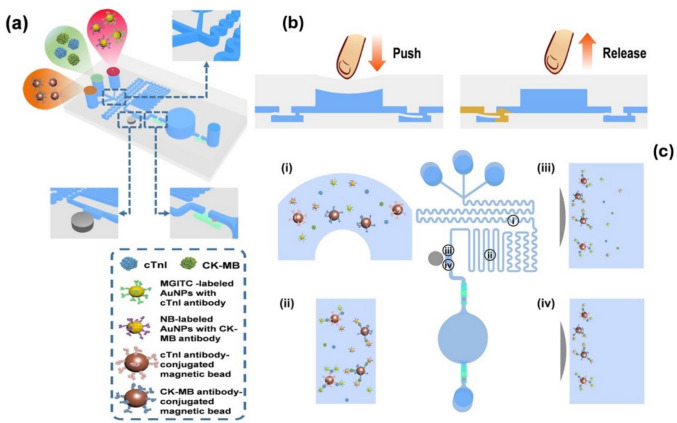
**a** The workflow of the SERS-based microfluidic chip with a finger-pump for simultaneous detection of two cardiac biomarkers. **b** Lateral view of the microfluidic chip with finger-pump and the schematic diagram of the check valves during the pressing and releasing actions of the finger-pump. **c** Schematic representation of the SERS-based immunoassay processes in the microfluidic channel. Adapted with permission from (Liu et al. 2023a), copyright (2022), Elsevier

A SERS-based sandwich immunoassay was developed by integrating a microfluidic chip with two channels in parallel (200 μm width × 100 μm depth) and an Au film-coated nanostripe microcone array substrate for the simultaneous detection of cTnI and CK-MB (Gao et al. 2022b). The main features of the proposed SERS substrate include its flexibility, large specific surface area to accommodate more sandwich immunocomplexes, and the possibility of generating more plasmonic hot spots. A mixture of cTnI and CK-MB antigens spiked into serum was introduced through inlet I at a fixed flow rate, and the corresponding SERS nanoprobes (MGITC and antibody-conjugated AuNPs) were simultaneously introduced through inlets II and III, each at a constant flow rate. The resulting sandwich immunocomplexes were formed on the antibody-conjugated SERS substrate. The characteristic peak of RRM MGITC at 1614 cm^−1^ was used as a reference to quantify the levels of cTnI and CK-MB. The entire immunoassay process was completed within 30 min, including fluid injection, immune reaction, washing with buffer, and SERS measurements. Liu et al. developed a microfluidic chip with a single-track finger-pump design, enabling the simultaneous SERS-based detection of cardiac biomarkers cTnI and CK-MB (Liu et al. 2023a). This microfluidic platform employed a sandwich immunoassay approach, integrating SERS nanoprobes (MGITC-labelled AuNPs conjugated with cTnI antibodies) and magnetic beads functionalized with cTnI antibodies. The microfluidic chip (60 mm × 30 mm) was composed of four components: a winding channel for immunoreactions, a collection microchamber for collecting immunocomplexes, check microvalves for reagent delivery, and a finger-operated pump for driving fluid flow through the channel. Figure 10 illustrates the workflow of the microfluidic chip with a finger-pump design, and schematics of the immunoassay process within the microfluidic channel for SERS-based simultaneous detection of dual cardiac biomarkers. The detection process includes: (1) injection of SERS nanoprobes, antibody-functionalized magnetic beads, and a mixture of cTnI and CK-MB into each of the three inlets; (2) thorough mixing of the biomarker and antibody solutions at position #i in the serpentine channel; (3) formation of immunocomplexes at #ii; (4) attraction of the immunocomplexes by the permanent round magnet, and collection in the detection chamber at #iii; and (5) washing away of unbound SERS nanoprobes with PBS buffer, followed by detection at position #iv. The main feature of this strategy is that the entire detection process is completed within 5 min, without a bulky syringe pump.

Paper-based analytical devices are effective platforms for POCT due to their cost efficiency, customizable shapes, simplicity, and compact design, which ensures portability (Pradela-Filho et al. 2023). Furthermore, the paper material has inherent capillary action, allowing for effortless fluid flow without the need for external pumping (Noh and Phillips 2010). Depending on the materials used and design approaches, paper-based devices can be classified into microfluidic paper-based analytical devices (μPADs) and lateral flow assays (LFA) (Chen et al. 2023a). In 2007, Whitesides and his colleagues first introduced the µPAD as a platform for bioassays (Martinez et al. 2007). µPADs are fabricated by patterning paper and typically contain hydrophobic barriers, hydrophilic channels, and detection zones (Nishat et al. 2021). The working mechanism of µPADs involves hydrophobic boundaries acting as physical barriers, guiding the sample flow along defined pathways within the hydrophilic regions. Distinct detection zones of µPADs are coated with specific receptors designed to identify the target analyte. The solutions move along the patterned pathways of filter paper by passive capillary action, reaching the detection zones for the simultaneous analysis of multiple targets (Sriram et al. 2017). LFAs are a type of POCT method, first introduced in 1984 as a urine-based pregnancy test (Majdinasab et al. 2022). The LFA is termed a lateral flow immunoassay (LFIA) when antibodies are used as the biorecognition elements. Compared to techniques like ELISA, LFIAs offer the advantages of minimal sample preparation and significantly shorter signal readout times (Kim et al. 2019). A typical LFIA test system consists of four main elements: (1) sample pad (SP): serves as the site for dropping the sample fluid. In some cases, the SP is pre-treated with surfactants and buffer salts to facilitate efficient sample flow and interaction throughout the test strip. In some assays, sample pretreatment is also performed in the SP. (2) Conjugate pad (CP): contains antibodies specific to the target analyte, most commonly conjugated to colloidal Au or latex microspheres. (3) Reaction membrane (usually nitrocellulose membrane): serves as a site for both the reaction and detection processes. It consists of a test line (T-line) and a control line (C-line), both of which are immobilized with capturing molecules (e.g., antibodies). Detection of the target analyte generates a signal at the T-line, while the signal at the C-line confirms proper sample flow through the strip. (4) Absorption pad (AP): positioned at the end of the strip, it wicks excess liquid sample and prevents backflow. All four elements are fixed onto an adhesive backing card to provide better stability and handling (Koczula and Gallotta 2016; Tripathi et al. 2018; Kim et al. 2019; Di Nardo et al. 2021; Pedreira-Rincón et al. 2025). Although paper-based devices have seen significant advancements, they generally provide dichotomous results, indicating only whether the analyte is present or absent. Gaining quantitative information can greatly enhance their efficiency and broaden their practical applications (Urusov et al. 2019). This can be achieved by integrating paper-based devices with SERS, which enables sensitive and quantitative detection of target analytes.

**Fig. 11:**
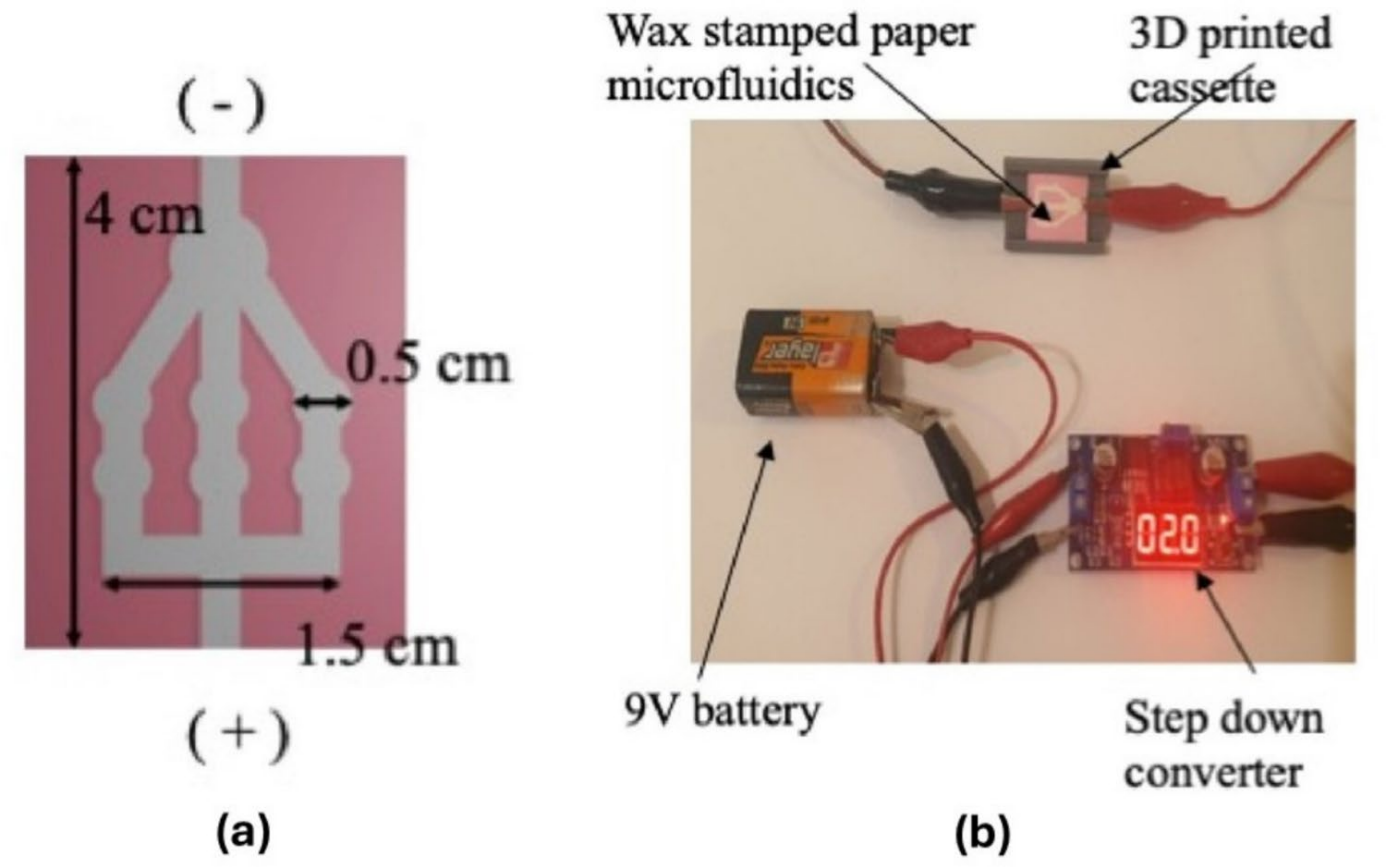
**a** Paper microfluidics.** b** Electric-modulated SERS detection setup. Adapted with permission from (Low et al. 2025), copyright (2024), Elsevier

Chemometrics is a chemical discipline that applies mathematics, statistics, and computer science to extract the most relevant information from complex chemical data (Héberger 2008). These techniques include multivariate analysis methods such as principal component analysis (PCA), partial least squares (PLS), multivariate curve resolution, cluster analysis, and discriminant analysis (Dos Santos et al. 2023). In SERS analysis, PCA and PLS are widely used for dimensionality reduction, feature extraction, to accurately classify samples and quantify analytes in complex spectral data (Shahzad et al. 2022). Lim et al. employed chemometrics techniques and a calibration-free approach for the simultaneous quantitative detection of multiple cardiac biomarkers, including cTnT, by integrating a µPAD with multiple reaction zones and SERS detection (Lim et al. 2020). In the calibration-free approach, the prediction of unknown analyte concentrations is based on the entire Raman spectrum, and the PLS method is employed. For cTnT detection, Au NPs@methyl red@silica nanotags functionalized with polyclonal anti-cTnT antibodies were used as SERS probes. These probes formed a sandwich immunocomplex with monoclonal anti-cTnT antibodies immobilized in the designated reaction zone of the µPAD. A SERS-based detection technique integrated into a µPAD platform was developed, incorporating electrical modulation and electromigration of unbound SERS tags (without multiple washing steps) for the simultaneous multiple detection, including cTnI, in human serum (Low et al. 2025). In this study, Au@4-nitroaniline@Ag@monoclonal cTnI antibodies form a sandwich immunocomplexes with the capturing antibodies immobilized in the detection zone in the presence of cTnI in the sample. In the control zone, chicken anti-mouse IgG secondary antibodies were deposited to capture unbound detecting antibodies functionalized on the SERS nanotags. The µPAD and electric field-modulated SERS detection setup is illustrated in Fig. 11. Electrophoresis was performed by placing the µPAD inside a cassette and running it for 5 min. To minimize electrophoresis time, the distance and size of the detection and control zones were optimized. All SERS measurements were performed under electrical modulation conditions, maintained at a constant voltage of 3 V. Artificial intelligence-based feature selection and dimensionality reduction were employed to interpret and analyze high-dimensional SERS spectra. Machine learning analysis of cTnI spectra using the Ridge regression model yielded the lowest root mean squared error (RMSE) of 4.375 × 10^–4^.

**Fig. 12:**
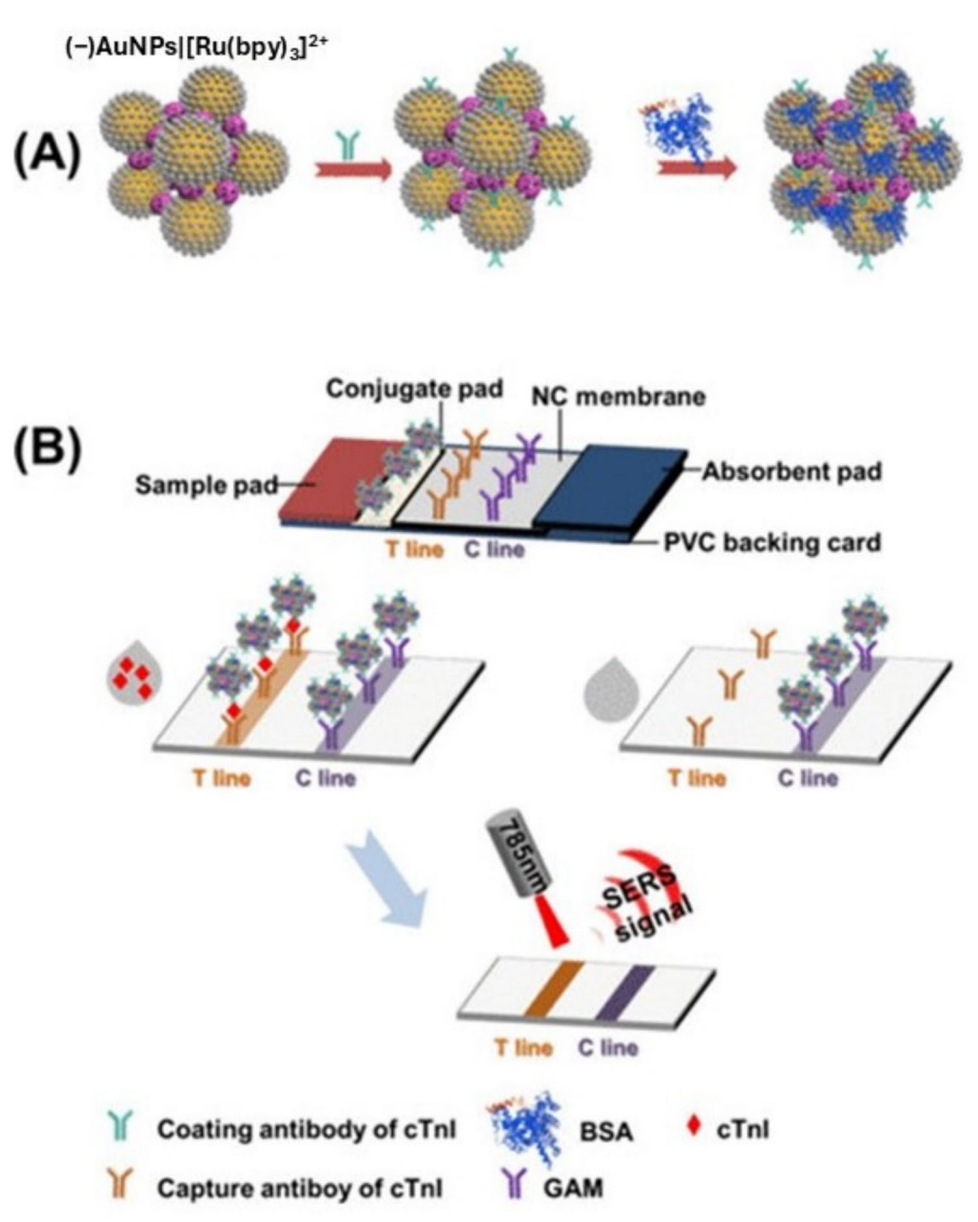
**A** Schematic diagram of the preparation of SERS immunoprobe. **B** Detection procedure. Adapted with permission from (Shao et al. 2024), copyright (2024), American Chemical Society

The use of SERS tags in LFIA has been investigated as a novel detection platform since 2007 (Doering et al. 2007). Currently, the SERS-LFIA platform is emerging as a promising tool for POCT applications (Sloan-Dennison et al. 2022). A SERS-based LFIA was developed for the quantitative detection of cTnI using gap-enhanced Raman tag (GERT), which consists of Au nanorod coated with an Au shell and incorporates 1,4-nitrobenzenethiol (NBT) molecules confined within a 1-nm gap between the core and shell (Khlebtsov et al. 2019). The working principle of the developed SERS-LFIA setup is as follows: Upon addition of the analyte solution to the SP, it flows to the CP, where cTnI specifically binds to anti-cTnI IgG conjugated with GERTs. The resulting complexes then migrate along the strip and bind to the antibodies on the T-line. At the same time, the excess conjugates continue to flow and are captured by the secondary antibodies on the C-line. At the end of the process, a color change in the test zone indicates the presence of the target analyte, which is then quantitatively analyzed using Raman microscopy mapping of the same zone. A 400 μm × 400 μm area inside the test zone was scanned, recording 1600 pixels in total, with an image acquisition time of approximately 15 min. The SERS images were decoded to obtain the characteristic peak intensities at 1296 and 1334 cm^−1^ for the quantitative analysis of cTnI. In another study, Song et al. primarily focused on optimizing various parameters of the SERS-based LFIA for cTnI detection, including AuNP size, MGITC concentration, LFIA components, fluid flow time, laser wavelength, and laser power (Song et al. 2023). Their study showed that by optimizing parameters, the LOD of the SERS-based LFIA reached 0.2 ng/mL, while that of the optical intensity-based LFIA was 1.56 ng/mL. Wang et al. reported a visual and quantitative LFIA for the simultaneous detection of dual biomarkers, cTnI and NT-proBNP, on separate T-lines (Wang et al. 2024a). In this study, polystyrene microspheres (PS) were functionalized with amino groups, enabling Au nanoparticles to be assembled onto their surface through electrostatic adsorption. Subsequently, SERS immunoprobes were synthesized by functionalizing cTnI antibodies onto 4-ATP-Au@PS particles. For the preparation of the LFIA, cTnI antibodies were sprayed onto T-line 2, and a nitrocellulose membrane with a pore size of 15 μm was selected. Different concentrations of cTnI samples were prepared in inactivated newborn calf serum. After the entire lateral flow process, the sandwich structure formed at each T-line provides both visual and SERS-based quantitative determination of the biomarkers. The developed LFIA demonstrates a 10–20-fold increase in sensitivity over commercial colloidal gold and fluorescent test strips for detecting the same biomarkers. Cross-reactivity between cTnI and NT-proBNP in the test strip was negligible. Recently, Shao et al. reported a study in which positively charged RRM trisbipyridine ruthenium (II) ions and negatively charged Au NPs combine through electrostatic interaction to form a SERS nanotag ((−) AuNPs|[Ru(bpy)_3_]^2+^) within 3 min to detect cTnI in human serum (Shao et al. 2024). The schematic diagram of the preparation procedure for the SERS immunoprobe and the detection method of cTnI is shown in Fig. 12. Following the same LFIA process described above, a sandwich immunocomplex forms at the T-line when cTnI is present in the sample, resulting in a dark-blue color at the T-line, with the color becoming deeper as the cTnI concentration increases. The remaining SERS immunoprobe binds to the GAM at the C-line, resulting in a dark-blue line that indicates the validity of the test. After 5min of the complete reaction, SERS measurements were performed on the T-line using a portable Raman spectrophotometer. The SERS intensity at 1041 cm^−1^ was utilized to construct the calibration curve for cTnI quantification. cTnI spiked into human serum samples was detected, with recovery rates ranging from 85.4% to 108.4%. The total assay time was 5 min, and the LOD was calculated to be 60pg/mL. In another study, a photostable GERT-based SERS-LFIA was developed for cTnI detection (Jin et al. 2025). The GERT is composed of Au NPs and NBT, forming a core–shell nanostructure. The antibody-conjugated GERT forms immunocomplexes at the T-line of the strip, resulting in a blue color visible to the naked eye. To meet POCT requirements, a portable Raman detector was used instead of Raman microscopy. A 4 mm × 1 mm area on the T-line was selected for analysis, and spectral data were collected from over 30 positions. Full-spectrum analysis was performed to obtain intensity values for each region. Signal curves of the samples were then plotted, and the area under the curve was estimated to determine the detection signal for each sample. The developed LFIA test showed a strong correlation with the chemiluminescence test when applied to clinical serum samples. Table 1 presents a summary of SERS-based immunoassays and apta assays for cTn detection.

**Table 1:** Summary of SERS-based immuno (apta) assays for cTn detection

LOD (ng/ml)	LR/DR (ng/ml)	Raman instrument	EW (nm)	RRM/Band marker (cm^−1^)	IT (min)	Specificity	Sample volume (μL)	Matrix	Validated method	Ref
0.005	0.01–1000	Renishaw inVia Raman microscope	633	MGITC/1613	180	IgG, PSA, CEA, glucose	10	SSM	…	Fu et al. 2019
0.0089	0.0–100	Renishaw inVia Raman microscope	632.8	MGITC/1615	120	IgG, HSA, BSA, Myo, CK	3	HS	CLIA	Cheng et al. 2019
0.0044	0.0–1.0	Raman spectrometer (Shanghai Ruhai Optoelectronics Technology)	…	4MP/1073.5	30	NT-proBNP, BSA, D-dimer	60	HS	CLIA	Hu et al. 2020
0.0098	0–2.0	Portable Raman spectrometer (Shanghai Ruhai Optoelectronics Technology)	…	4-MBA/1075	30	H-FABP, D-dimmer, NT-proBNP, BSA	50	HS	CLIA	Hu et al. 2021
0.0086	0.01–10	Laser Raman Microscope	633	4-MBA/1078	15	NT-proBNP, Myo, PCT, CK-BB, BSA	10	PBS	…	Wang et al. 2022
0.00316	0.01–100	PeakSeeker Pro 785E Raman spectrometer	785	DTNB/1333	120	Myo, CEA, AFP, HSA, IgG	15	Whole blood	…	Wang et al. 2021
0.00909	0.01–100	Renishaw, inVia™ confocal Raman microscope	785	4-MBA/1077	240	AFP, BSA, Horse Serum	50	…	…	Xiang et al. 2025
0.00001181	0.1–1000	Benchtop: UniDRON (UniNanoTech) Portable: Mira DS (Metrohm Ag)	785	2-NAT/1380	10	cTnT, CRP, BNP	…	HS	ELISA	Lee et al. 2024a
0.00001079	0.0001–10	Home-built Raman microscopy system	633	DTNB/1332	10	Myo, HCG, CK-MB, BSA, Phe, Lys, His, Tyr, Gly	5	serum	…	Ma et al. 2025
0.00033	0.001–50	BWTek i-Raman Plus (USA)	785	4-MP/1077	60	CEA, PSA, PDGFB, hIgG	25	HS	CMIA	Zhao et al. 2023
0.0058	0.001–1000	Confocal micro-Raman spectrometer (WEVE, HEDA)	633	MGITC/1615	60	…	…	…	…	Yoo et al. 2025
0.0049	0.01–100	Laser Confocal Raman Microspectroscopy system	532	CBBG/1612	70	AchE, BSA, CC, COD	100	HS	ELISA	Qin et al. 2024
10	…	Renishaw InVia Reflex confocal Raman spectrometer	785	Label free	…	…	10	PBS	…	Alves et al. 2020
0.0055	0.01–100	XploRAPLUS Raman Microscope (HORIBA)	638	CBBG/1621	70	IgG, HSA, CEA, LPL, AChE	100	HS (commercial)	…	Lin et al. 2022b
0.0024	0.0024–2.4	Homemade micro-Raman	633	Cy5/1580	420	TnC, TnT, IgG, avidin	1000	HS	ELISA	Lee et al. 2020
0.00023	0.001–100	XpoloRAPLUS Raman Microscope (HORIBA)	638	1471	110	AchE, BSA, HSA, IgG, LPL	100	HS	…	Lin et al. 2022a
0.00027	0.001–100	Renishaw inVia microRaman spectrometer	785	4-MBA/1585	120	CEA, AFP, PSA, BSA	10	HS	ELISA	Wang et al. 2024b
0.0001	0.0001–10	Laser confocal Raman spectrometer	785	2-MPY/998	120	IgG, EGF, CRP, IL-2	20	HS	CLIA	Wu et al. 2025
0.01	0.01–50	LabRam HR evolution system (HORIBA)	632.8	MGITC/1614	…	CK-MB, BSA, Thrombin, PSA	…	Serum	…	Gao et al. 2022a
0.01	0.01 − 1000	LabRam HR Evolution system (HORIBA)	633	MGITC/1614	30	CK-MB, BSA, Thrombin, PSA	15	Serum (synthetic)	…	Gao et al. 2022b
0.00504	0.01–1000	LabRAM HR Evolution system (HORIBA)	632.8	MGITC/1614	5	…	70	HS	…	Liu et al. 2023a
0.001	0.001–200	Renishaw InVia Raman Microscope	514	methyl red/entire spectrum	9	…	10	HS	Siemens Centaur XPT IA	Lim et al. 2020
0.02464	0.0016–16	TechnoSpex µ-Raman-Ci Raman Micro-Spectroscopy system	532	4-nitroaniline/1129	…	…	100	HS	Siemens Alletica IA	Low et al. 2025
0.1	0.03–100	InVia Renishaw confocal Raman microscope	785	NBT/1280–1360	10	…	70	…	…	Khlebtsov et al. 2019
0.02	0.01–100	Horiba Xplora Plus microRaman	638	MGITC/1614	15	CRP, Myo, Tau, BSA	…	HS	…	Song et al. 2023
0.001	0.001–40	…	785	4-ATP/1090	15	cTnC, cTnT, sTnI	100	INCS	…	Wang et al. 2024a
0.060	0.7–75	Portable Raman spectrophotometer	785	[Ru(bpy)_3_]^2+^/1041	5	AFP, HCG, PSA, CEA, Glucose, Urea, Vitamin C	50	HS	CL	Shao et al. 2024
0.00065	0.0025–8	portable Raman Spider2000 +	785	NBT/entire spectrum	15	…	65	HS	Beckmann CL	Jin et al. 2025

*LR/DR* linear range/detection range, *EW* excitation wavelength, *IT* immunoreaction time, *IgG* human immunoglobulin G, *PSA* prostate specific antigen, *CEA* carcino-embryonic antigen, *HSA* human serum albumin, *BSA* bovine serum albumin, *Myo* myoglobin, *CK* creatine kinase, *PCT* procalcitonin, *CK-BB* creatine kinase–brain isoenzyme, *AFP* alpha-fetoprotein, *HCG* chorionic gonadotropin, *CRP* C-reactive protein, *BNP* brain natriuretic peptide, *CK-MB* creatine kinase MB isoform, *Phe* phenylalanine, *Lys* lysine, *His* histidine, *Tyr* tyrosine, *Gly* glycine, *LPL* lipoprotein lipase, *AChE* acetylcholinesterase, *TnC* troponin C, *TnT* troponin T, *sTnI* skeletal troponin I, *PDGFB* platelet-derived growth factor subunit B, *CC* cytochrome *C,* *COD* cholesterol oxidase, *EGF* epidermal growth factor, *IL-2* interleukin-2, *PBS* phosphate buffered saline, *SSM* serum substitute medium, *HS* human serum, *INCS* inactivated newborn calf serum, *CLIA* chemiluminescence immunoassay, *IA* immunoassay, *CL* chemiluminescence, *CMIA* chemiluminescence microparticle immunoassay

## Summary and outlook

Early diagnosis of CVD is critically important for the effective treatment and recovery of patients. Therefore, the development of simple, rapid, and ultra-sensitive diagnostic tools for CVD, particularly AMI, is essential for detecting cardiac biomarkers at minute concentrations in biological fluids. Troponin is widely recognized as the gold-standard biomarker for diagnosing AMI, with several analytical techniques established for its sensitive and specific detection. Although the sensitivity and accuracy of POCT devices may not yet match those of centralized laboratory tests, they remain at the forefront of cTn detection due to continuous advancements and integration of innovative technologies. The main features of POCT include high sensitivity, specificity, rapid results, along with the other REASSURED criteria. Herein, we discuss the key aspects of SERS-based assays and their feasibility as a POCT platform for cTn detection. (1) These assays demonstrated sufficient analytical sensitivity for cTn detection, with LODs ranging from a few ng/mL to even fg/mL. However, the 99th percentile value of the test is required to fully assess its clinical performance (Low et al. 2020). (2) Since cTn has a low Raman cross section, label-based assay appears to be an effective strategy for the quantitative and multiplexed detection across a broad range of sample concentrations. (3) Due to its high specificity, the sandwich immunoassay format is commonly employed in SERS-based detection. (4) Noble metals such as Au and Ag are primarily used to fabricate SERS substrates. Graphene and graphene oxide have also demonstrated excellent SERS activity, either independently or in combination with noble metals. Plasmonic NPs with various geometries, such as spherical, core-shell, and nanocubes, are used to fabricate SERS nanotags. GERTs can generate intense EM hotspots within the nanogap, while also protecting the RRMs from external conditions and preventing NPs aggregation. (5) In what we previously referred to as traditional SERS platforms, Raman measurements are carried out either in solution or on the surface of a static substrate. If the solution mode is selected, Raman measurements can be conducted in the same container where the immunoassay reaction occurs. However, this approach may suffer from lower experimental reproducibility due to the continuous movement of the plasmonic nanoparticles in solution. An improvement to this approach involves the use of magnetic beads or magnetic NPs, which can be immobilized on the container or substrate using an external magnet to perform Raman measurements. A static substrate can be glass or plasmonic materials. A dual plasmonic enhancement can be achieved when the capture substrate consists of an array of plasmonic nanostructures. (6) The disadvantages of the above platforms include a lack of precise control over sample volume, flow rate, and reaction conditions. Additionally, the washing, mixing, and separation procedures inherent to the analytical process further increase the complexity of the approach. These limitations can be addressed by integrating the SERS detection strategy with microfluidic and paper-based platforms. Automation is one of the requirements for POCT, and the integration of microfluidic platforms can meet this criterion, enabling effective multiplexed detection. Microfluidic systems can operate in both passive and active modes, each offering distinct advantages and limitations. (7) SERS-based LFIA can meet POCT requirements such as low costs and rapid results (within a few minutes). These systems do not require complex instrumentation, and integrating LFIA with portable Raman devices enhances the potential for on-site detection. However, SERS-based LFIAs often require careful optimization and handling to ensure reproducibility, as the Raman signal can be influenced by flow dynamics and the physical properties of the nitrocellulose membrane. The nitrocellulose membrane used in LFIA strips is inherently heterogeneous, leading to variability in Raman intensities both within an intra-assay and between inter-assays. (8) The use of antibodies as biorecognition elements in SERS-based assays is attributed to their specific antigen–antibody interactions, making them a preferred choice for detecting cTn. A multi-antibody assay offers additional advantages in terms of improved sensitivity and accuracy. A variety of antibodies are available for cTn detection, including monoclonal, recombinant, and polyclonal antibodies. However, the optimal combination for use in SERS-based immunoassays has not yet been established. While designing immunoassays for troponin detection, several challenges should be taken into consideration, including cross-reactivity between antibodies and troponin isoforms, masking of troponin binding sites by the troponin complex, proteolytic degradation of the terminals (N and C) on cTnI, and assay interference by anti-species antibodies in human (Ma et al. 2021). A potential approach to overcome the inherent limitations of antibodies is the use of aptamers. However, aptamer-based assays for troponin detection are still in the growing stages of development; further research is needed to validate their advantages and limitations. (9) In SERS-based assays, calibration is commonly used to quantify analyte concentrations. The narrow linear dynamic range of the calibration curve is not ideal for POCT applications, as it may limit accurate detection across clinically relevant concentrations. To address this issue, calibration-free approaches that utilize statistical methods can be effective. Machine learning is an effective tool for feature selection from spectral data and classification in the context of multiplexed detection. Building predictive and classification machine learning models with minimal errors offers a promising approach for achieving rapid and accurate POC diagnosis. (10) Benchtop Raman instruments are widely used in research and can be adapted for small clinical setups. As discussed, portable Raman devices are more suitable for POCT applications in some respects. However, the high cost of both benchtop and portable systems remains a significant barrier for widespread use. Developing an affordable Raman setup without compromising the sensitivity and accuracy of detection could greatly enhance the accessibility and future potential of troponin diagnosis at the POC. (11) Studies have confirmed the presence of cTn in saliva (Mishra et al. 2018; Domenico et al. 2023). But their detection using SERS has not yet been reported. Exploring this approach is of great significance, as it holds the potential to establish a non-invasive SERS-based method for troponin detection.

## Data Availability

No datasets were generated or analysed during the current study.
